# Cysteine-Directed Isobaric Labeling Combined with GeLC-FAIMS-MS for Quantitative Top-Down Proteomics

**DOI:** 10.1021/acs.jproteome.4c00835

**Published:** 2025-01-31

**Authors:** Theo Matzanke, Philipp T. Kaulich, Kyowon Jeong, Ayako Takemori, Nobuaki Takemori, Oliver Kohlbacher, Andreas Tholey

**Affiliations:** † Systematic Proteome Research & Bioanalytics, Institute for Experimental Medicine, Christian-Albrechts-Universität zu Kiel, 24105 Kiel, Germany; ‡ Applied Bioinformatics, Computer Science Department, University of Tübingen, Sand 14, 72076 Tübingen, Germany; § Institute for Bioinformatics and Medical Informatics, University of Tübingen, Sand 14, 72076 Tübingen, Germany; ∥ Translational Bioinformatics, University Hospital Tübingen, Hoppe-Seyler-Str. 9, 72076 Tübingen, Germany; ⊥ Advanced Research Support Center, Institute for Promotion of Science and Technology, Ehime University, Toon 791-0295, Japan

**Keywords:** FAIMS, isobaric labeling, proteoform, top-down proteomics, quantification, SDS-PAGE

## Abstract

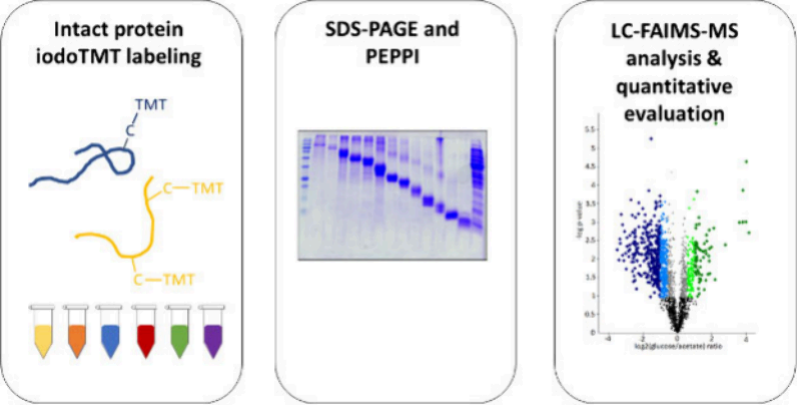

The quantification of proteoforms, i.e., all molecular forms in which proteins can be present, by top-down proteomics provides essential insights into biological processes at the molecular level. Isobaric labeling-based quantification strategies are suitable for multidimensional separation strategies and allow for multiplexing of the samples. Here, we investigated cysteine-directed isobaric labeling by iodoTMT in combination with a gel- and gas-phase fractionation (GeLC-FAIMS-MS) for in-depth quantitative proteoform analysis. We optimized the acquisition workflow (i.e., the FAIMS compensation voltages, isolation windows, acquisition strategy, and fragmentation method) using a two-proteome mix to increase the number of quantified proteoforms and reduce ratio compression. Additionally, we implemented a mass feature-based quantification strategy in the widely used deconvolution algorithm FLASHDeconv, which improves and facilitates data analysis. The optimized iodoTMT GeLC-FAIMS-MS workflow was applied to quantitatively analyze the proteome of *Escherichia coli* grown under glucose or acetate as the sole carbon source, resulting in the identification of 726 differentially abundant proteoforms.

## Introduction

Numerous biological processes during the transcription (e.g., mRNA-splicing) and during or after translation can lead to the formation of manifold protein molecules, namely proteoforms,^1^ of which the number exceeds that of the genes by far. The various modifications lead to a steep increase in proteome complexity, with the different proteoforms having different physicochemical properties and biological functions. Thus, the identification and quantification of proteoforms is an essential prerequisite to understanding biological processes at the molecular level.

Proteomics approaches can be classified into two major strategies: bottom-up (BUP) and top-down proteomics (TDP). In BUP, proteins are digested into peptides, which are then analyzed, e.g., by liquid chromatography–mass spectrometry (LC-MS), and assigned to protein groups by employing protein inference algorithms. While BUP approaches are well-elaborated and highly sensitive, they lead to a loss of proteoform information.^1,2^ In contrast, TDP is based on intact proteoform analysis and, thus, retains the entire proteoform information. However, a limitation of TDP is the inherently lower sensitivity when analyzing large proteoforms compared to small peptides identified by BUP. With the increasing size, the number of isotopes and charge states increases, splitting the overall signal intensity and, thus, decreasing the overall sensitivity.^3^ Besides that, problems of TDP are encountered, e.g., by the chromatographic separation of large proteoforms and the high spectral complexity at the MS1 and MS2 levels, resulting in overlapping peaks. Due to these reasons, TDP currently faces an upper mass limit of approximately 30–35 kDa.^3,4^

In all proteomics approaches, efficient separation of the analytes prior to MS is a major step to cope with sample complexity. In this respect, in the early days of proteomics, GeLC-MS^5^ had become a workhorse technology in BUP. This method combines intact proteoform level separation by sodium dodecyl sulfate-polyacrylamide gel electrophoresis (SDS-PAGE), followed by an in-gel digestion of the proteins, peptide elution, and subsequent LC-MS-based analytics at the peptide level.

The application of GeLC-MS for TDP was, for a long time, hampered by low recovery rates of intact proteoforms from polyacrylamide gels. The development of PEPPI-MS (Passively Eluting Proteins from Polyacrylamide gels as Intact species for MS)^6^ helped to overcome this hurdle. With its ability to prefractionate and isolate proteoforms in different mass ranges, easily achieved by cutting the gel in distinct mass zones prior to elution, the PEPPI approach showed particularly beneficial in combination with FAIMS utilizing internal compensation voltage stepping^3,7−9^ (GeLC-FAIMS-MS).

For quantitative analysis of proteoforms, label-free quantification (LFQ) has been applied in numerous TDP approaches.^10−12^ Limiting factors are long instrument times due to the need to analyze a sufficient number of replicates, and the combination with multidimensional separation schemes is critical due to variations in sample preparation.^13^ Recently, a proteoform reaction monitoring approach has been developed, enabling the targeted quantification of proteoforms.^14,15^

Isobaric labeling^16^ enables multidimensional separation and minimizes the instrument time required, however, is still underrepresented in TDP due to several challenges. Amino-directed isobaric labeling of intact proteins^17^ can suffer from factors such as potential over- and under-labeling, leading to a large number of species out of a given proteoform^18^ and is accompanied by a number of challenges in data interpretation.^19^ Despite these challenges, several recent studies employed amino-directed isobaric labeling for TDP quantification.^20−22^

An alternative approach recently introduced as a viable alternative for quantitative TDP is Cys-directed isobaric labeling using iodo-tandem mass tags (iodoTMT).^23^ While iodoTMT inherently excludes proteoforms not containing cysteine from the quantification, current limitations in database search and labeling efficiency present in aminoTMT are omitted with this approach.^19^

**Figure 1:**
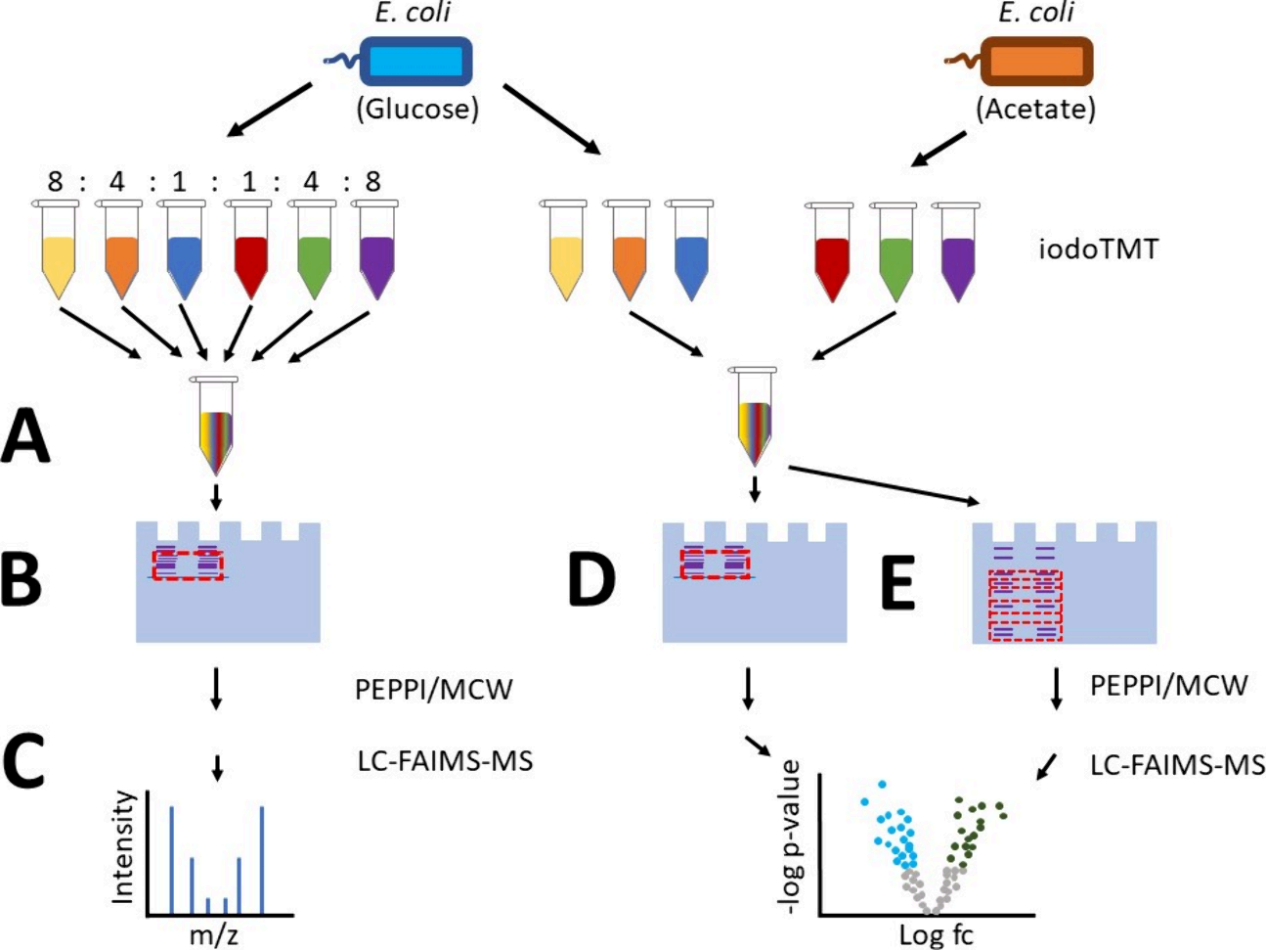
Study design for evaluating the isobaric labeling-based GeLC-FAIMS-MS approach for proteoform-level quantification of *E. coli* proteomes. (A) Lysate of the same biological replicate was labeled with six different iodoTMT-reagents and mixed in defined ratios (8:4:1:1:4:8). (B) After SDS-PAGE separation, gel pieces in the range ≤40 kDa were collected and proteoforms extracted using PEPPI in conjunction with methanol-chloroform-water precipitation (MCW). (C) The resulting sample was used to assess the quantitative accuracy of the workflow and MS method benchmarks. (D, E): Three biological replicates from *E. coli* grown on glucose or acetate minimal medium were lysed and labeled using iodoTMT-sixplex and combined. (D) Proteoforms were isolated and extracted by PEPPI/MCW from a single gel band stretching the mass range smaller than approximately 40 kDa (referred to as one PEPPI-fraction). (E) Ten gel slices were excised in the ≤40 kDa mass range and extracted by PEPPI/MCW (referred to as ten PEPPI fractions) using an extrusion-tip protocol^25−27^ to assess the benefit of sample fractionation.

In this study, we explored the combination of cysteine-directed isobaric labeling with the GeLC-FAIMS-MS approach (Figure 1). The quantitative accuracy of the workflow was evaluated using a defined ratio of iodoTMT sixplex-labeled *Escherichia coli* proteome. We optimized the GeLC-FAIMS-MS/MS workflow (e.g., fragmentation strategy, isolation width, and the application of FAIMS with internal CV stepping) to increase the number of quantified proteoforms and reduce ratio compression. A mass feature-based quantification method was integrated into the FLASHDeconv deconvolution software,^24^ which allows standardized TMT reporter ion detection for large data sets. With the optimized GeLC-FAIMS-MS workflow and data analysis pipeline, the relative proteoform abundances in *E. coli* grown on either glucose or acetate as the sole carbon source were analyzed.

## Materials and Methods

### Materials

Unless stated otherwise, all chemicals and solutions were purchased from Sigma-Aldrich (Steinheim, Germany). Ultrapure water (18.2 MΩ/cm^–1^) was prepared using the Arium VF 611 system (Sartorius, Göttingen, Germany).

### *Escherichia coli* Cultivation

*Escherichia coli* K-12 strain MG1655 was cultured as described previously.^28^ Briefly, bacteria were cultured in M9 minimal medium^29^ supplemented with glucose (15 mM) or acetate (45 mM) as the sole carbon source. Bacteria were inoculated at an optical density at 600 nm (OD_600_) of 0.1 and incubated at 37 °C under constant shaking. The cells were harvested at an OD_600_ of approximately 1 by centrifugation for 3 min at 3,000 rcf and room temperature. The cells were washed with ultrapure water and stored at −80 °C prior to cell lysis.

### Cell Lysis

Frozen aliquots of *E. coli* cells were resuspended in 1 mL of 8 M guanidine hydrochloride, 200 mM triethylammonium bicarbonate (TEAB, pH 8.5), 1× cOmplete EDTA-free protease inhibitor (Roche, Basel, Swiss) and lysed using an ultrasonic source (Bandelin electronics, Berlin, Germany). Samples were cooled on ice during sonication in ten cycles in 30 s (Power 28%). Cellular debris was removed by centrifugation at 21,100 rcf for 30 min at 4 °C, and the protein concentration was determined utilizing the Pierce BCA Protein assay kit (Thermo Scientific, Waltham, MA).

### Intact Protein IodoTMT Labeling

Cys-directed iodoTMT labeling was conducted to label all proteoforms directly after cell lysis, as described previously.^23^ In brief, 160 μg protein was transferred into a reaction tube for each TMT channel, and the volume was filled up to 80 μL with lysis buffer. For proteoform reduction, 1 μL 200 mM Tris(2-Carboxyethyl)-phosphine (TCEP) was added and incubated for 60 min at 50 °C and 800 rpm on a shaker. The iodoTMTsixplex reagents (Thermo Scientific) were resuspended in 10 μL methanol and added to the samples, which were then incubated for 50 min at 37 °C and 800 rpm in the dark. The reaction was quenched by adding 4 μL of 200 mM DTT and incubation for 15 min at room temperature. The samples were combined prior to purification by methanol-chloroform water precipitation. Prior to proteoform separation by SDS-PAGE, the protein pellets were resuspended in Laemmli buffer^30^ and heated for 15 min at 70 °C.

For a proof of principle experiment, one biological *E. coli* replicate (grown under glucose condition) was divided into six aliquots and labeled with iodoTMT sixplex using a defined ratio of 8:4:1:1:4:8 (126:127:128:129:130:131) (Figure 1A). For the quantitative proteoform analysis of *E. coli* grown under glucose or acetate as the sole carbon source, three biological replicates each were labeled with different iodoTMT channels (channels 126–128 for the glucose and channels 129–131 for the acetate samples) (Figure 1 D-E).

### SDS-PAGE

For gel electrophoretic separation, a 16% separation gel (2.1 mL ultrapure water, 5.3 mL 30% acrylamide/Bis, 2.5 mL 1.5 M Tris-HCl pH 8.8, 100 μL 10% (w/v) SDS, 50 μL 10% (w/v) ammonium persulfate (APS), 10 μL tetramethylethylenediamine (TEMED)) in combination with a 4% stacking gel (2.1 mL ultrapure water, 1.3 mL 30% acrylamide/Bis, 2.5 mL 0.5 M Tris-HCl pH 6.8, 100 μL 10% (w/v) SDS, 50 μL 10% (w/v) APS, 10 μL TEMED) was cast using the PROTEAN 1 mm system (BioRad, Hercules, CA). Each sample was loaded into two adjacent gel pockets, with 80 μg loaded for the enrichment of proteoforms smaller than 40 kDa and 100 μg for the fractionation approach. The proteoforms were separated using an electric field at a constant voltage of 50 V, and after the running front reached the separation gel, the voltage was increased to 90 V. The electrophoretic separation was monitored with the Pierce Prestained Protein Ladder (Thermo Scientific). For enriching proteoforms smaller than 40 kDa, the electrophoresis was terminated after the 40 kDa marker band was separated from other marker bands (Figure 1B, D). In contrast, for fractionating the proteoforms in 10 fractions, the electrophoresis was performed until the running front reached the end of the gel (Figure 1E).

After electrophoresis, the gels were incubated for 20 min in fixing solution (40% (v/v) methanol, 5% (v/v) acetic acid), and the proteoforms were stained for 30 min using Coomassie staining solution (0.2% (w/v) Coomassie R250, 0.05% (w/v) Coomassie G250, 10% (v/v) ethanol, 40% (v/v) methanol, 2% (v/v) glycerin). Fixation of the gel was conducted as it has been shown to increase the recovery of small proteoforms.^6^ The gels were destained in a destaining solution (10% (v/v) ethanol, 5% (v/v) acetic acid) overnight.

### Passive Protein Extraction from the Gel

The proteoforms were extracted from the gel using the polyacrylamide-gel-based prefractionation for analysis of intact proteoforms (PEPPI) protocol as previously described.^6^ In brief, the gels were washed with water before the gel regions of interest of two adjacent gel lanes were excised with a scalpel and transferred into a 1.5 mL reaction tube. The gel pieces were homogenized for 1 min using a BioMasher plastic pestle (Nippi, Tokyo, Japan), and 300 μL of 0.1% (w/v) SDS, 100 mM ammonium bicarbonate (ABC, ∼pH 9) were added, followed by 30 s of further homogenization.

Alternatively, for the fractionation of the sample into ten fractions, an extrusion-tip protocol^25−27^ was employed to crush the gel pieces and parallelize the extraction. Extrusion-tips were manufactured by cutting the top of a GELoader tip (Eppendorf, Hamburg, Germany), so that it could be inserted into a 200 μL tip (Suppl. Figure S1). Ten fractions below 40 kDa were excised and subjected to the extrusion-tips placed in 2 mL tubes. Extrusion was conducted by centrifugation for 4 min at 21,100 rcf. Then, 300 μL of 0.1% SDS (w/v), 100 mM ABC (∼pH 9) were added.

For passive elution of the proteoforms from the gel matrix, the samples were shaken for 30 min at 1,500 rpm and room temperature. The gel pieces were removed using a Spin-X centrifugal filter (45 μm pore size, cellulose acetate, Corning, Corning, NY, USA) by 3 min centrifugation at 3,000 rcf. The filtrate was subjected to methanol-chloroform-water precipitation to purify the proteoforms.

### Methanol-Chloroform-Water Precipitation

Methanol-chloroform-water (MCW) precipitation was performed for proteoform purification as described previously.^8^ In brief, the sample was transferred to a 1.5 mL reaction tube, and if necessary, the volume was adjusted to 300 μL with ultrapure water. Then, 600 μL of methanol, 150 μL chloroform, and 400 μL ultrapure water were added with thorough vortexing between each solvent addition. The upper layer was removed after centrifugation at 14,000 rcf for 10 min. Next, 600 μL of methanol was added, and the tube was gently inverted prior to centrifugation (10 min, 14,000 rcf). The supernatant was removed, and the pellet was washed twice with 600 μL methanol. After the second washing, the pellet was dried for 30 min in a fume hood. For MS analysis, the protein pellet was resuspended in 20 μL 3% acetonitrile (ACN), 0.05% trifluoroacetic acid (TFA), vortexed, briefly sonicated, and centrifuged for 5 min at 21,100 rcf. The supernatant was transferred into LC-MS vials with glass inserts and stored at −80 °C until LC-MS analysis.

### LC-MS/MS

Chromatographic separation of proteoforms was conducted using a Dionex U3000 UHPLC system equipped with a C4 analytical column (50 cm × 75 μm, 2.6 μm, 150 Å, Thermo Fisher) and a C4 precolumn (C4 PepMap300, 5 μm, 300 Å, Thermo Fisher). The following 120 min gradient was employed using eluent A (0.05% formic acid (FA)) and eluent B (80% ACN, 0.04% FA) at 300 nL/min and 45 °C: 0–5 min 4% B, 5–7 min 4–15% B, 7–127 min 15–50% B, 127–129 min 50–90% B, 129–140 min 90% B, 140–150 min 90–4% B. One μL of the sample were injected into the LC, corresponding to approximately 0.5–1 μg of protein.

The LC was coupled online to a Fusion Lumos Tribrid mass spectrometer (Thermo Scientific, Bremen, Germany) equipped with a FAIMS Pro interface (Thermo Scientific). Each sample was injected twice, using two complementary multi-CV-FAIMS-MS/MS methods to cover a wide proteoform mass range.^7^ For targeting proteoforms smaller than approximately 20 kDa, a high/high acquisition strategy and more negative CVs were applied (hereinafter referred to as high/high method). In contrast, a medium/high acquisition^31^ and more positive CVs were applied to target larger proteoforms (medium/high method). The MS settings were optimized for certain CVs based on the favored proteoform mass range.^7^ Within a cycle time of 3 s, the most intense precursors (charge states 4–50, including undetermined charge states; dynamic exclusion was enabled with *n* = 2, *t* = 60 s) were selected for fragmentation. Two fragmentation strategies were investigated in this study: (i) A stepped HCD fragmentation (30, 40, 50%), referred to as the one-scan method, and (ii) two separate MS/MS events (CID 25%, HCD 80%) for proteoform identification and TMT quantification, referred to as the two-scan method.

All details of the MS settings (resolutions, AGC target, ion injection time) utilized for the different methods and FAIMS CVs are listed in Supporting Table S1.

### Deconvolution, Reporter Ion Extraction, and Database Search

Raw files were converted to the mzML format using msConvert,^32^ with peak picking enabled for the high/high data acquired with high MS1 resolution. Deconvolution and mass feature TMT ratio extraction were performed with FLASHDeconv (version 3.1.0-pre-FDdevelop-2024–08–17).^24^ Briefly, while performing spectral deconvolution, FLASHDeconv also measures reporter ion intensities using IsobaricQuantifier library in OpenMS.^33^ Then FLASHDeconv aggregates the reporter ion intensities in MS2 spectra from the same deconvolved precursor masses (subject to input ppm mass tolerance) within 30 s retention time window to increase the signal-to-noise ratio of the quantification while reducing missing values. This aggregated quantification information is hereafter referred to as “mass-feature-based quantification”.

Default deconvolution parameters were used, except for the medium/high data (low MS1 resolution), where the MS1 tolerance was increased to 50 ppm. The generated TopFD output files were analyzed with TopPIC^34^ (v.1.6.5) against a FASTA protein database containing proteins from *E. coli* K12 (taxon: 83333, UniProt release 2022_03). TopPIC database search settings were set as default if not mentioned otherwise. IodoTMT sixplex was set as a fixed modification (329.226595 Da, cysteine residues) and acetylation (42.0106 Da, lysine residues), phosphorylation (79.9663 Da, serine, threonine, and tyrosine residues) and oxidation (15.9949 Da, methionine, cysteine, lysine, aspartate, arginine, asparagine, and tyrosine residues) were allowed as variable PTMs with the maximum number of variable PTMs set to three. A maximum number of two unexpected modifications within a mass shift of ±500 Da were allowed, enabling the identification of both previously known and unknown modified proteoforms. For each quantification method, the identification results from replicates were merged into a single result, keeping an overall proteoform-level false discovery rate (FDR) of 1%. To do so, we keep all the target and decoy identifications in each TopPIC analysis, allowing FDR of 100%. When merging, the proteoforms of the same sequence and precursor masses (within 10 ppm mass errors) were merged into a single hit, with its E-value score being the lowest (i.e., the best) E-value of the merged proteoforms. Then, FDR was calculated with these merged proteoforms, and only the ones with FDR less than 1% were retained.

### Quantitative Analysis

Quantitative information from the FLASHDeconv output was assigned to the identified proteoforms via the scan numbers of the corresponding raw file. For classical quantitative analysis, the quantitative TMT reporter ion information on a single MS2 scan (“QuantForCh1–6” in FLASHDeconv output) was matched to the associated proteoform based on the scan number reported by TopPIC. Thus, each proteoform is only quantified by the MS2 scan related to the identification of the proteoform.

In contrast, for the mass feature-based quantification, the combined quantitative information on several quantification scans originating from the same mass feature (within a 10 ppm precursor mass and 30 s retention time tolerance) were combined (“MergedQuantForCh1–6” in FLASHDeconv output) and matched to the TopPIC search results via the scan number. This means that the mass feature-based quantification uses the quantitative information from all MS2 spectra acquired for a given mass feature, even if not all of them led to the identification of the proteoform. Proteoforms without cysteines or lacking one of the six TMT reporter ion intensities were removed prior to the quantitative analysis.

Statistical analysis was performed in Perseus^35^ (v.2.0.10.0). TMT reporter ion intensities were median normalized, log2 transformed, and assigned to their respective biological condition. A two-sided paired *t* test (s0 = 0, permutation-based FDR, cutoff displayed in plot, 250 randomizations) was performed to evaluate differentially abundant proteoforms.

### Data Availability

All raw data has been uploaded to the ProteomeXchange consortium^36^ via the PRIDE partner repository under the accession PXD055510.

## Results and Discussion

### Proof of Principle

To demonstrate the compatibility of iodoTMT labeling with the GeLC-FAIMS-MS workflow for the quantitative analysis of proteoforms by TDP, an *E. coli* lysate was divided into six aliquots, labeled with iodoTMTsixplex reagents and recombined at a defined ratio of 8:4:1:1:4:8 (Figure 1A). The sample was separated by SDS-PAGE and a gel slice below approximately 40 kDa (i.e., the mass range currently accessible for in-depth TDP^37^) was excised in a single fraction and proteoforms extracted using the PEPPI procedure. This approach leads to the depletion of large proteoforms but is not considered a fractionation approach in the traditional sense of a two-dimensional separation. The proteoforms were purified and analyzed by LC-FAIMS-MS/MS.

The labeling efficiency was examined by deconvolution of the raw files^24^ and mass shift analysis by MSTopDiff^38^ (Suppl. Figure S2). Besides the typical high abundant mass shifts at 16.00 Da (oxidation) and 32.00 Da (dioxidation), no mass shift assignable to iodoTMT over- or under-labeling was detected, demonstrating the high labeling efficiency and overall quality of the data.

For quantitative analysis, the reporter ion intensities were determined for each MS2 spectrum and assigned to the correlated proteoform by the scan number (in the following referred to as the “classical quantification approach”). Overall, 2,492 proteoforms were identified, of which 631 (25.3%) could be quantified, i.e., these proteoforms contain at least one cysteine in their sequence, and all six TMT reporter ions were detected.

**Figure 2:**
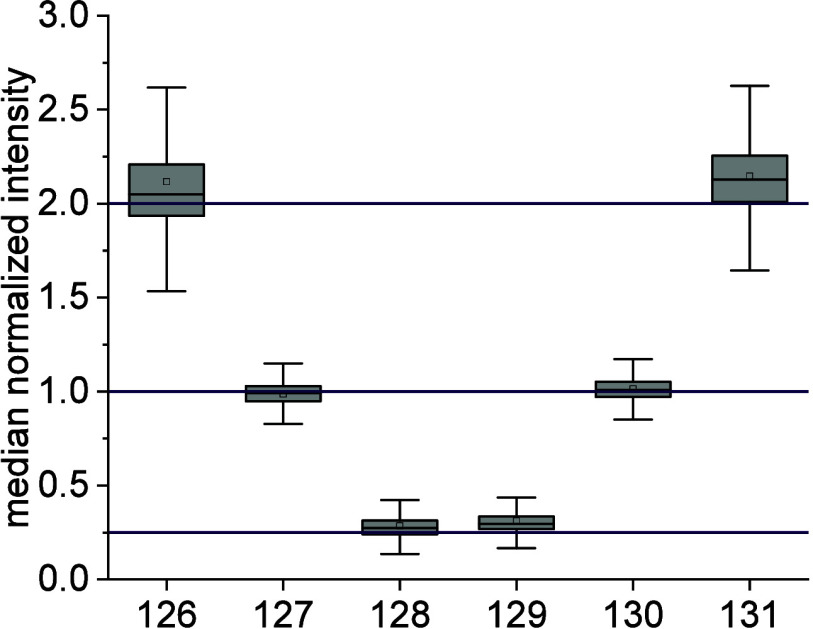
Mass feature-based TMT quantification of *E. coli* proteoforms labeled with iodoTMT and mixed in defined ratios (8:4:1:1:4:8). For the mass feature-based quantification, the reporter ion intensities of several quantification scans originating from the same mass feature (within a defined mass and retention time tolerance) were combined and matched to the proteoform identification. The reported proteoform quantity distributions after median normalization are shown, with the true ratios depicted as purple lines. The boxplot shows the 25th and 75th percentile, with the whiskers indicating the 1.5 interquartile range. The black lines represent the median, and the square points represent the average value.

In addition to this classical quantification approach, a new tool was implemented in the widely used deconvolution algorithm FLASHDeconv (version 3.1.0-pre-FDdevelop-2024–08–17) that enables quantification at the mass feature level (“mass feature-based quantification”) by combining the quantitative information (i.e., the intensities of the reporter ions) of MS2 spectra generated by the same precursor masses (subject to a mass tolerance) within a 30 s retention time window. Unlike the classical quantification, the mass feature-based approach has the advantage of utilizing quantitative information from MS2 spectra that may not necessarily result in the identification of proteoforms but contain reporter TMT ions. Both the classical and the mass feature-based approach resulted in a distribution of the TMT reporter ions (Figure 2) that approximate the expected ratios (8:4:1:1:4:8). However, the mass feature-based approach showed slightly narrower distributions around the expected values (Suppl. Figure S4). This observation can be explained by the high number of quantitative data used for the mass feature-based approach (on average, 2.5 quantitative fragment scans per precursor), resulting in a robust proteoform quantification, which is, e.g., less susceptible to effects such as coisolation and ratio compression.

It is noteworthy that, in some cases, TMT reporter ions were detected from precursors assigned to proteoforms lacking a cysteine residue, e.g., due to coisolation with cysteine-containing proteoforms or nonspecific reporter ion assignment. In order to verify whether the quantification algorithm assigns nonspecific reporter ions, we additionally analyzed a sample without TMT-labeled proteoforms. No TMT ratios were reported over the entire length of the gradient, demonstrating the accuracy of the reporter ion assignment and rules out artifacts introduced by the quantification algorithm. Thus, the occurrence of TMT reporter ions from precursors assigned to proteoforms without a cysteine residue can likely be explained by coisolation with cysteine-containing proteoforms.

**Figure 3:**
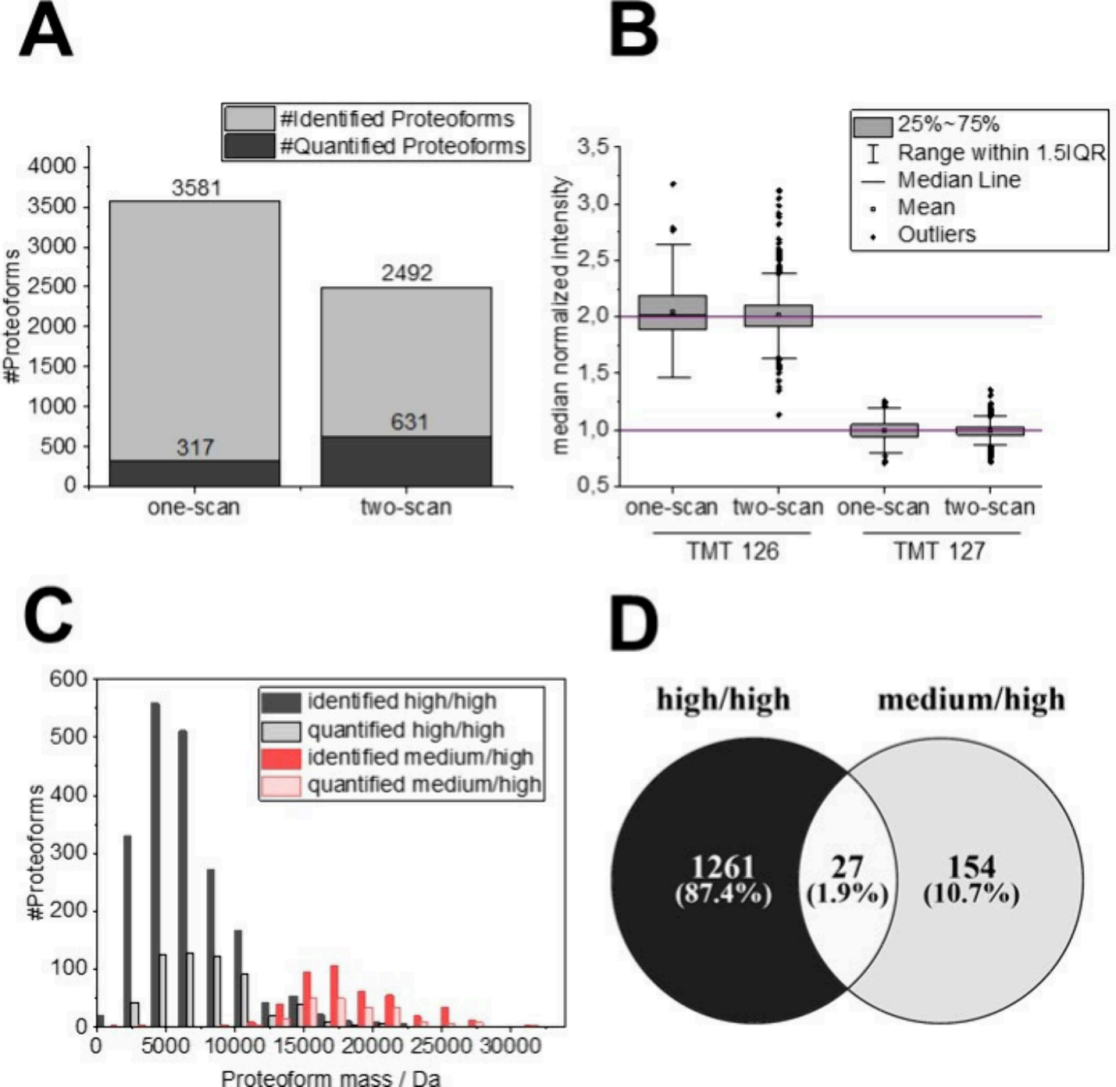
MS method optimization to increase the depth of the quantitative top-down analysis. (A) Number of identified and quantified proteoforms utilizing the one-scan (single scan with stepped HCD fragmentation) and the two-scan method (two separate MS2 scans for proteoform identification and quantification). (B) Quantification accuracy of the one- and two-scan methods for two selected TMT channels. (C) Mass distribution of the identified and quantified proteoforms using MS methods targeting low-molecular-weight proteoforms (more negative FAIMS CVs and high-resolution MS1 and MS2 scans, referred to as high/high MS method) and high-molecular-weight proteoforms (more positive CVs and low-resolution MS1 and high-resolution MS2 scans, medium/high). (D) Venn diagram of the proteoform sequences identified with the high/high and medium/high MS method.

The mass distribution of the quantified proteoforms was shifted toward higher molecular weights compared to that of all identified proteoforms (Figure 3C). This might be due to the higher stochastic abundance of cysteine residues with increasing proteoform sequence length, i.e., the longer the protein sequence, the higher the probability that a cysteine residue is present.

### MS Optimization

A major challenge in isobaric labeling-based approaches is that proteoform fragment generation and efficient TMT reporter ion formation require different fragmentation energies.^20,39^ Two main ion activation strategies have been utilized for labeling-based quantitative TDP: (*i*) HCD fragmentation with stepped collision energies (referred to as the one-scan method; here, we used HCD fragmentation with normalized collision energies of 30, 40 and 50%),^20^ and (*ii*) the performance of two separate fragmentation scans for identification and quantification, utilizing optimized collision energies for the two tasks (two-scan method; quantification scan with HCD and an NCE of 80% and identification scan with CID and an NCE of 25%).^23^

In order to optimize the LC-FAIMS-MS/MS setup, the same sample used in the proof of principle experiment was analyzed utilizing the two different MS/MS acquisition approaches. The two-scan method significantly outperformed the one-scan method in terms of the number of quantified proteoforms (631 and 317 quantified proteoforms, respectively, Figure 3A). This observation can be explained by the efficient generation of TMT reporter ions by the two-scan method at high collision energies, as revealed by the ratio of quantified to identified cysteine-containing proteoforms (58.3% for two-scan, and 26.7% for one-scan). However, more proteoforms were identified using the one-scan (3,581) compared to the two-scan method (2,492), as theoretically, every MS2 scan can result in the identification of a proteoform, while for the two-scan method, only every second spectrum can result in a proteoform identification.

The distribution of the experimentally determined TMT ratios was close to the expected values for both fragmentation methods (Figure 3B). Nevertheless, the one-scan method showed slightly broader distributions of the reporter ion ratios, which can probably be attributed to a lower number of quantitative data points, i.e., the number of utilized scans with detected reporter ions. Only 7.5% of MS2 spectra contained all TMT reporter ions for the one-scan method (an average of 674 spectra per raw file), while 39.5% of all HCD spectra contained all TMT reporter ions for the two-scan method (an average of 2228 spectra per raw file). Based on the presented results, the two-scan method was selected for all future experiments.

Another challenge in labeling-based quantification is the cofragmentation of multiple proteoforms caused by coeluting analytes, resulting in ratio compression (i.e., an underestimation of the proteoform abundance).^40^ Note that in iodoTMT-based quantification experiments, ratio compression is generally expected to be lower compared to aminoTMT-based experiments since the number of labeled analytes is much lower, reducing the likelihood of coisolation of labeled analytes and, thus, reducing the overall ratio compression.^23^

One possibility to diminish the effect of ratio compression is narrowing the isolation window to avoid cofragmentation of precursors with closely spaced isotopic envelopes.^41^ However, this decreases the sensitivity of TDP analysis as the larger isotopic distribution of proteoforms in comparison to peptides has to be considered.^42^ The sweet spot of the isolation window regarding the sensitivity and ion compression was examined using a two-proteome interference model with *E. coli* (TMT ratios 8:4:1:1:4:8) and yeast (4:4:4:4:4:4) proteoforms (Suppl. Figure S4A). The highest number of quantified proteoforms was obtained with an isolation window of 2 *m*/*z* (*n* = 317 *E. coli* proteoforms, Suppl. Figure S4B). In contrast, larger isolation windows resulted in a lower number of identifications (3 *m*/*z*: *n* = 284), e.g., due to extensive coisolation and the occurrence of chimeric spectra, hampering the subsequent data analysis.^42^ In addition, also smaller isolation windows resulted in a significantly lower number of quantified proteoforms (1.2 *m*/*z*: *n* = 223), likely due to the decreased sensitivity. The effect of ratio compression was slightly reduced when narrowing the isolation window from 3 *m*/*z* to 2 *m*/*z*; however, no further improvement was observed when using an isolation window of 1.2 *m*/*z* (Suppl. Figure S4C). Controlling the isolation window width dynamically based on signal-to-ratio within the window by advanced real-time data acquisition control (e.g., by FLASHIda^43^) could help to further reduce the ratio compression.

Another possibility to tackle the challenge of ratio compression is the fractionation of proteoforms to reduce the sample complexity prior to the MS analysis. In this study, we used the gas-phase fractionation technique FAIMS utilizing internal compensation voltage (CV) stepping. Notably, different CVs in FAIMS favor the identification of proteoforms within specific mass ranges,^44^ which is the base for a recently established workflow encompassing internal CV stepping,^7^ allowing the identification of proteoforms over a broad range of proteoform masses. Moreover, this approach enables to adapt the MS1 and MS2 parameters to the needs of specific proteoform mass ranges.^7,8^ We employed two complementary LC-multi-CV-FAIMS-MS methods to cover a broad proteoform mass range. The first method aimed to target lower mass proteoforms using more negative CVs and a high/high acquisition strategy, i.e., high-resolution MS1 and MS2 parameters, including adapted values for the number of microscans and injection times. In contrast, the second method aimed to target larger proteoforms by using more positive CVs and a medium/high acquisition strategy,^31^ i.e., medium-resolution MS1 scans but an elevated number of microscans and high-resolution MS2 parameters (Figure 3C). Compared to measurements without FAIMS, the multi-CV-FAIMS approach substantially improved the number of quantified proteoforms (317 vs 106 proteoforms) and reduced ratio compression, demonstrating that gas-phase fractionation efficiently reduces spectral complexity^44^ (Suppl. Figure S4B,C). Moreover, the complementarity in the number of identified proteoforms also led to a higher number of quantified proteoforms (Figure 3D). While a direct comparison with other TDP data sets is hampered by the fact that different sample preparation protocols may lead to different proteoform identifications^45^ and depend strongly on factors such as the MS and data interpretation algorithms utilized, the mass distribution of the proteoforms identified in our study (10.35 (mean) ± 6.42 (standard deviation) kDa) was comparable with that reported for *E. coli* (11.41 ± 6.77 kDa)^46^ and for a collection of different bacteria (7.49 ± 5.11 kDa).^47^

In summary, the combination of iodoTMT-labeling, gel-based separation, PEPPI-based extraction of the proteoforms, and FAIMS with internal CV stepping, as well as a two-scan method using optimized fragmentation energies for the generation of proteoform fragments and reporter ions and a new mass feature-based data analysis tool integrated in FLASHDeconv enables an optimized in-depth quantitative analysis of proteoforms by TDP.

### Quantitative Top-Down Analysis of *E. coli* Grown on Different Carbon Sources

The optimized GeLC-FAIMS-MS/MS workflow was applied to quantitatively analyze the proteome of *E. coli* cells cultivated in a minimal medium supplemented with glucose or acetate as the sole carbon source, respectively.^28^ Three biological replicates for each condition were labeled with different channels of the iodoTMT sixplex. The combined samples were processed using (*i*) a single gel band containing proteoforms smaller than 40 kDa (Figure 1D) and (*ii*) ten fractions of proteoforms smaller than 40 kDa (Figure 1E). A protocol that combines the principles of the ConGnaC-tip,^25^ a Gel Shredder^26^ and Syringe Maceration Extraction,^27^ was utilized to homogenize the gel pieces by extruding them through a narrow-cavity pipet tip via centrifugation in order to process multiple samples simultaneously (Suppl. Figure S1).

**Figure 4:**
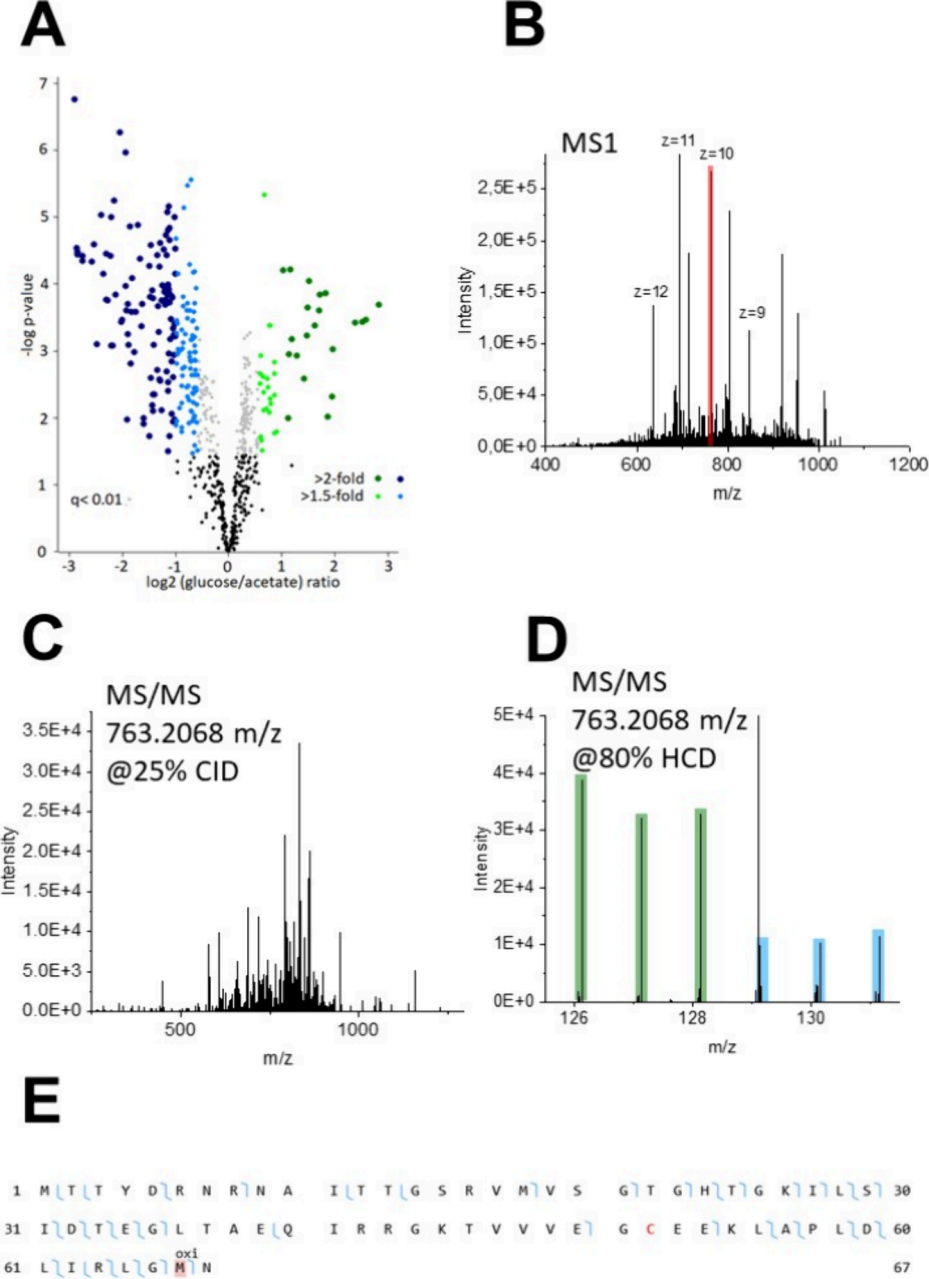
Quantitative TDP analysis of the proteome of *E. coli* grown under glucose and acetate as the sole carbon source using a single PEPPI fraction. (A) Volcano plot showing the quantified proteoforms, with 324 highlighted proteoforms identified as significantly differentially abundant. (B–E) One oxidized proteoform of the putative selenoprotein YdfZ was significantly differentially abundant under glucose growth conditions. (B) MS1 spectrum with the isolation window (shown in red), (C) MS2 identification scan (25% CID), (D) MS2 quantification scan (80% HCD) with the TMT reporter ions reflecting the abundances under glucose and acetate growth conditions highlighted in green and blue, respectively. In (E), the fragment map of the proteoform is shown.

Analyzing three technical replicates with the high/high and medium/high methods, 2,426 proteoforms were identified in the single PEPPI fraction, of which 784 proteoforms (32.1%) from 137 proteins could be quantified. From these, 324 proteoforms showed a fold change of at least 1.4 and, thus, were classified as significantly differentially abundant (Figure 4A).

**Figure 5:**
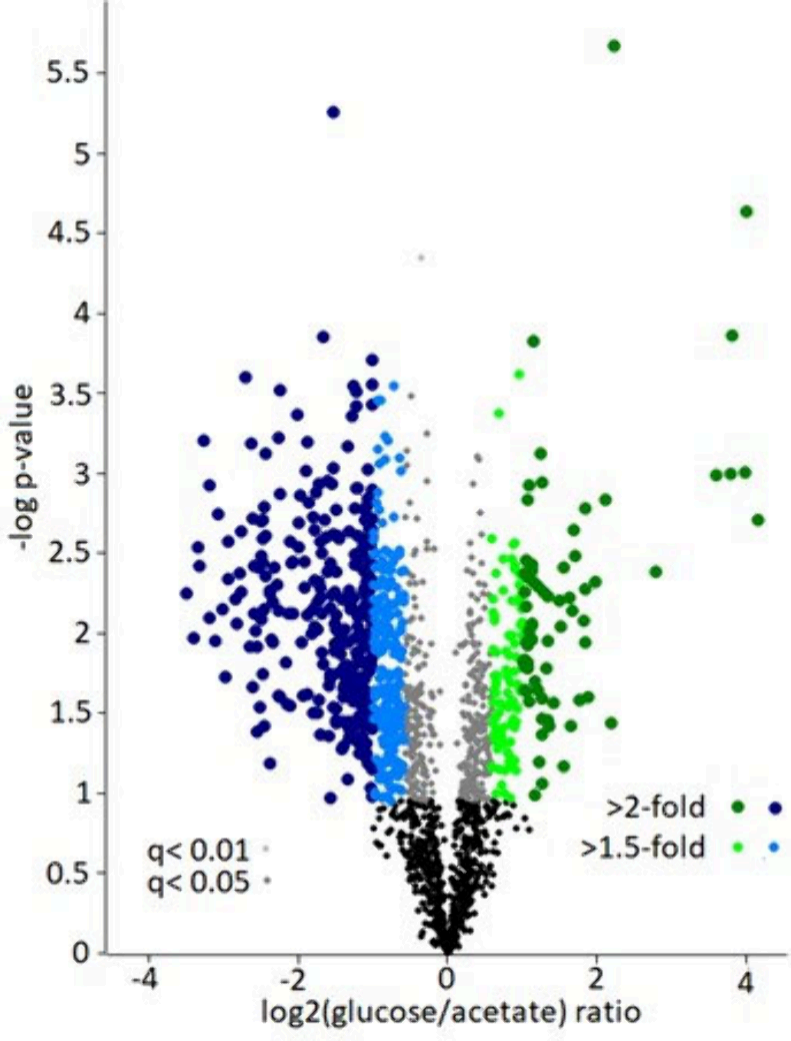
Proteoform-level changes in the proteome of *E. coli* in dependence on the carbon source (glucose or acetate) as revealed by the LC-FAIMS-MS analysis of 10 PEPPI fractions <40 kDa. A total of 726 proteoforms were found to be significantly differentially abundant between the two biological conditions.

In contrast, the multidimensional separation using ten gel fractions led to a significantly increased number of identified (4,964 proteoforms) and quantified (1,614 = 34.1%) proteoforms (Suppl. Figure S5A). However, it should be noted that the greater depth of analysis required a significantly longer measurement time (112.5 instead of 15 h). With increasing molecular weight, the relative proportion of quantified proteoforms increased (Suppl. Figure S5B). In total, 726 proteoforms from 140 proteins were significantly differentially abundant (fold change ≥1.4). From these, 172 proteoforms (from 46 proteins) were significantly more abundant under glucose conditions, and 554 proteoforms (from 95 proteins) under acetate conditions (Figure 5, Suppl. Table S2–S25).

A variety of proteoforms were quantified that are involved in carbohydrate metabolic processes, such as triosephosphate isomerase (P0A858), glyceraldehyde-3-phosphate dehydrogenase A (P0A9B2), or fructose-bisphosphate aldolase class A (P0AB71). Furthermore, various ribosomal proteoforms were quantified. The overall highest fold change was observed for six C-terminally truncated proteoforms from the autonomous glycyl radical cofactor (P68066, log2 fold-change larger than 3.5), which were significantly higher abundant in the glucose condition. Another example of a proteoform that was significantly higher abundant under glucose growth conditions (log2 fold-change larger than 3) was the putative selenoprotein YdfZ (P64463) (Figure 4B–E).

Multiple examples of proteins were detected, of which various proteoforms were identified, but only specific proteoforms showed differing abundance, including some with opposite abundance. For example, the Succiante-CoA ligase (ADP-forming) subunit alpha (P0AGE9) was identified with 16 proteoforms. However, mainly modified full-length and C-terminally truncated proteoforms were significantly higher abundant under acetate growth conditions (log2 fold change −1.72), whereas the N-terminally truncated proteoforms were not significantly differentially abundant, indicating potentially different biological functions (Suppl. Figure S6A, B). The same was observed for the Succiante-CoA ligase (ADP-forming) subunit beta (UniProt accession P0A836), the other part of the functional unit in the TCA cycle. Two N-terminally truncated proteoforms showed insignificant abundance changes, while five other truncated proteoforms were significantly higher abundant under acetate conditions (Suppl. Figure S6C, D).

Quantification at the proteoform level by top-down proteomics can elucidate changes in the proteome that are difficult to detect with classical BUP-based quantitative proteomics.^12^ Two quantified proteoforms of the selenide, water dikinase (P16456) were significantly differentially abundant, one in each of the two compared biological conditions (Suppl. Figure S7A–G). Both proteoforms cover the same canonical protein sequence; however, the proteoform enriched under acetate-supplemented growth conditions showed a mass shift of −281.243 Da, which could be explained as a trioxidation instead of an iodoTMT tag on Cys17 (theoretical mass shift of −281.2418 Da, proteoform Δ*M* = −0.24 ppm). The proteoforms may have different biological functions as indicated by their different abundance and are difficult to separate in a BUP experiment as most tryptic peptides are identical between the two proteoforms. Indeed, in a BUP study of the same biological conditions,^28^ the protein was not significantly differentially abundant (log2 fold-change of 0.36), highlighting the utility of quantitative TDP.

## Conclusion

In this study, we developed a workflow for quantitative in-depth TDP encompassing cysteine-directed isobaric labeling combined with GeLC-FAIMS-MS and subsequent data analysis using FLASHDeconv. We demonstrated the general compatibility of the gel-based fractionation approach, PEPPI, with iodoTMT quantification. The labeling-based approach allowed for the multiplexing of the analysis of up to six samples in parallel. Compared to other TDP sample preparation workflows for isolating proteoforms such as filter- or depletion-based methods,^45^ PEPPI allows for fractionation, substantially improving the number of quantified proteoforms. Note that we utilized MCW precipitation for proteoform purification; however, other purification methods are applicable based on the specific research question, e.g., to enhance the recovery of small proteoforms.^8,11,48^

The MS settings were optimized to address the most common challenges in labeling-based quantitative proteomics: fragmentation of the precursors, i.e., to ensure sufficient reporter ion generation and proteoform fragment ions, and ratio compression caused by the cofragmentation of multiple proteoforms. A method utilizing separate MS2 scans optimized for quantification (i.e., applying high collision energy of 80% HCD) and identification (25% CID) resulted in a significant increase in the number of quantified proteoforms compared to a one-scan method applying stepped HCD (30/40/50%) fragmentation. The ratio compression was successfully reduced by narrowing the isolation width to 2 *m*/*z*, which is the sweet spot of diminishing cofragmentation and maintaining sensitivity. Additionally, gas-phase fractionation with FAIMS significantly improved the number of quantified proteoforms and reduced ratio compression compared to measurement without FAIMS due to the decreased spectral complexity. We used complementary LC-FAIMS-MS methods utilizing FAIMS with internal CV stepping, targeting the lower and higher mass proteoforms, significantly increasing the number of identifications across a wide proteoform mass range.

A newly implemented tool in the FLASHDeconv deconvolution software enables mass feature-based quantification of isobaric labeling-based TDP data. FLASHDeconv combines the quantitative information on multiple MS2 spectra generated by the same precursors (within a certain mass and retention time tolerance), resulting in a more robust quantification. The mass feature-based quantification is independent of the proteoform identifications since they are matched by scan number or mass. Thus, this approach is compatible with all database search engines. Notably, all common labeling strategies (iTRAQ4plex, iTRAQ8plex, TMT10plex, TMT11plex, TMT16plex, TMT18plex, TMT6plex) are already integrated into FLASHDeconv and can be analyzed, ensuring simple and reproducible quantification and eliminating the need for in-house (often unpublished) scripts.

The optimized GeLC-FAIMS-MS workflow was utilized to quantitatively analyze the proteomes of *E. coli* cells grown under glucose or acetate as the sole carbon source. Fractionation into 10 fractions with PEPPI resulted in the quantification of 1,614 proteoforms (from 288 proteins), from which 726 proteoforms were significantly differently abundant. Recently, an LFQ-based quantification on a similar model system has been published, where proteoforms below 30 kDa were enriched with PEPPI and analyzed using LC-FAIMS-MS.^11^ Although more expensive and technically more challenging, iodoTMT offers the advantage of multiplexing, resulting in a reduced overall measurement time (i.e., higher throughput) and a lower number of missing values compared to LFQ. Moreover, labeling the sample directly after cell disruption and combining the labeled samples in the early stage of sample preparation reduces the sampling bias. As multidimensional fractionation schemes are essential for in-depth TDP studies,^49^ the straightforward combination with isobaric labeling enables comprehensive proteoform identification.

An inherent limitation of iodoTMT labeling is that only cysteine-containing proteoforms can be quantified. Approximately 84% of proteins in the *E. coli* proteome contain at least one cysteine;^50^ however, in our study, only about 1/3 of the identified proteoforms could be quantified. We observed a larger proportion of quantified proteoforms as their mass increased, likely due to the increased stochastic probability of containing a cysteine with increasing sequence length. Due to the advantages over aminoTMT, such as almost no overlabeling observed, iodoTMT may be particularly suitable for large proteoforms. However, should the present limitations still encountered with aminoTMT labeling of intact proteins (under/and overlabeling, and database search) be solved in the future, the presented workflow here is certainly applicable, too. Currently, the multiplexing capability of iodoTMT is limited to six channels; however, the commercial release of higher multiplexed reagents could further expand the application of this approach.

Compared to a previously published quantitative BUP study of *E. coli* with the same growth conditions,^28^ the presented TDP provided quantification information on the proteoform level. We identified changes in the abundance of proteoforms, which have been masked by the peptide-based protein quantification using BUP due to the need for protein inference. Thus, TDP, even if much less sensitive and hampered by the limitations regarding the currently accessible mass range mentioned above, adds an important layer of complexity to elucidate the processes in the cell on a molecular level since different proteoforms of the same protein can have different functions.

In summary, we here presented a workflow for in-depth quantitative top-down proteomics, including cysteine-targeting iodoTMT labeling, GeLC-FAIMS-MS analysis, and mass feature-based quantification. The integration of the data analysis into the widely used deconvolution software FLASHDeconv enables the easy evaluation of label-based TDP experiments. Due to the flexibility of the PEPPI approach regarding the number and complexity of the analyzed fractions and the specific mass range, a suitable quantitative TDP approach can be designed based on certain research questions.

## Supporting Information Available

Preparation of extrusion-tips for PEPPI-MS (Suppl. Figure S1); evaluation of iodoTMT labeling efficiency using MSTopDiff (Suppl. Figure S2); Accuracy of the mass feature-based quantification using FLASHDeconv (Suppl. Figure S3); impact of FAIMS on TMT ratio compression and proteoform identification (Suppl. Figure S4); fractionation increases the depth of the quantitative TDP analysis (Suppl. Figure S5); Evaluation of different succinate-CoA ligase subunit proteoforms (Suppl. Figure S6); analysis of selenide, water dikinase proteoforms (Suppl. Figure S7); detailed MS and FAIMS settings used in this study (Suppl. Table S1) (PDF)
List of all identified and quantified proteoforms from all data sets in this study (Suppl. Table S2–S25) (XLSX)
