# Supplementary material for: Cysteine-Directed Isobaric Labeling Combined with GeLC-FAIMS-MS for Quantitative Top-Down Proteomics
Source: J Proteome Res. 2025 Jan 31;24(3):1470–80. doi: 10.1021/acs.jproteome.4c00835 (PMC11894657; doi:10.1021/acs.jproteome.4c00835)
Supplement: Supplementary file 1 — pr4c00835_si_001.pdf [file pr4c00835_si_001.pdf]

Supplementary Information:

Cysteine-directed isobaric labeling combined with GeLC-FAIMS-MS for quantitative top-down proteomics

Theo Matzanke[1], Philipp T. Kaulich[1], Kyowon Jeong[2,3], Ayako Takemori[5], Nobuaki Takemori[5], Oliver Kohlbacher[2,3,4], and Andreas Tholey[1]*

[1]Systematic Proteome Research & Bioanalytics, Institute for Experimental Medicine, Christian-Albrechts-Universität zu Kiel, 24105 Kiel, Germany

[2]Applied Bioinformatics, Computer Science Department, University of Tübingen, Sand 14, 72076 Tübingen, Germany

[3]Institute for Bioinformatics and Medical Informatics, University of Tübingen, Sand 14, 72076 Tübingen, Germany

[4]Translational Bioinformatics, University Hospital Tübingen, Hoppe-Seyler-Str. 9, 72076 Tübingen, Germany

[5]Advanced Research Support Center, Institute for Promotion of Science and Technology, Ehime University, Toon, Japan

Contents

Supplementary Figures 2

Suppl. Fig. 1: Preparation of extrusion-tips for PEPPI-MS.......... 2

Suppl. Fig. 2: Evaluation of iodoTMT labeling efficiency.......... 3

Suppl. Fig. 3: Accuracy of the mass feature-based quantification. 4

Suppl.Fig. 4: Impact of FAIMS on ratio compression and proteoform identification. 5

Suppl. Fig. 5: Fractionation increases the depth of the quantitative TDP analysis. 6

Suppl. Fig. 6: Evaluation of different succinate-CoA ligase subunit proteoforms.......... 7

Suppl. Fig. 7: Analysis of selenide, water dikinase proteoforms.......... 8

References.......... 9

Supplementary Tables 10

Suppl. Table 1: MS and FAIMS settings used in this study. 10

Supplementary Tables 2-25: see separate Excel file, lists of identified and quantified proteoforms

Supplementary Figures

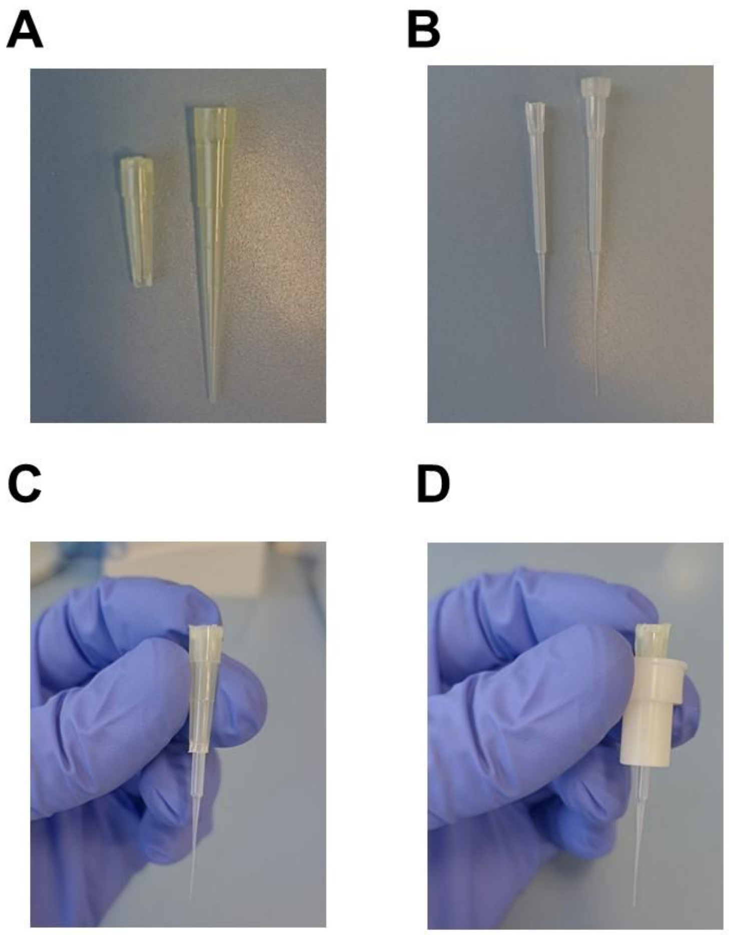

Suppl. Fig. 1: Preparation of extrusion-tips [1–3] for PEPPI-MS. (A) 200 µL tips were cut as an adapter and a funnel to fit the GELoader tip while allowing larger gel pieces to be loaded. (B) The GELoader tip was cut so that the tip did not touch the bottom of the tube once installed while maintaining the narrow cavity. (C) The GELoader tip was then inserted into the 200 µL tip and (D) fitted to a 2 mL tube using a stage-tip adapter.

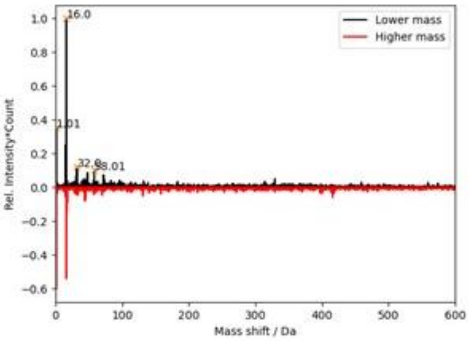

Suppl. Fig. 2: Detection of frequently occurring mass shifts to assess the quality of the labeling. After deconvolution by FLASHDeconv [4], MSTopDiff [5] analysis was performed (default settings, intensity×count plot, bin size 0.01 Da). No significant over- or underlabeling was observed (mass shift of 329.23 Da with bin size of 0.01 Da). The most dominant signals can be assigned to deconvolution error (1.01), oxidation (16.00), and di-oxidation (32.00).

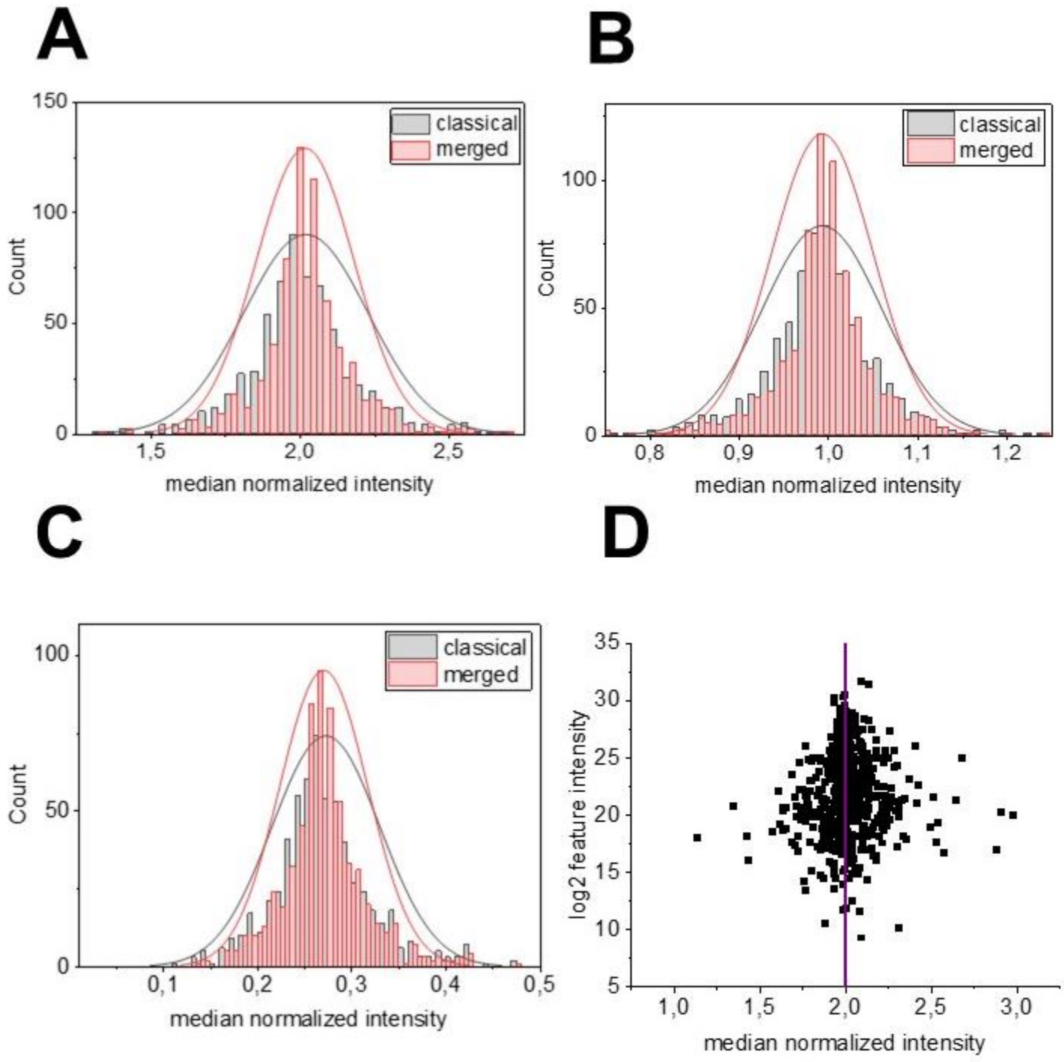

Suppl. Fig. 3: Comparison of the mass feature-based and the classical quantification strategy. For channels (A) 126, (B) 127, and (C) 128, the distributions of the normalized intensities are slightly closer to the true value when the mass feature-based quantification strategy is used, and the distribution is narrower compared to the classical quantification approach. (D) The accuracy for the TMT quantification is largely independent of the proteoform abundance as the true ratio value of 2 (purple line) is approximated across different orders of magnitude of feature intensity.

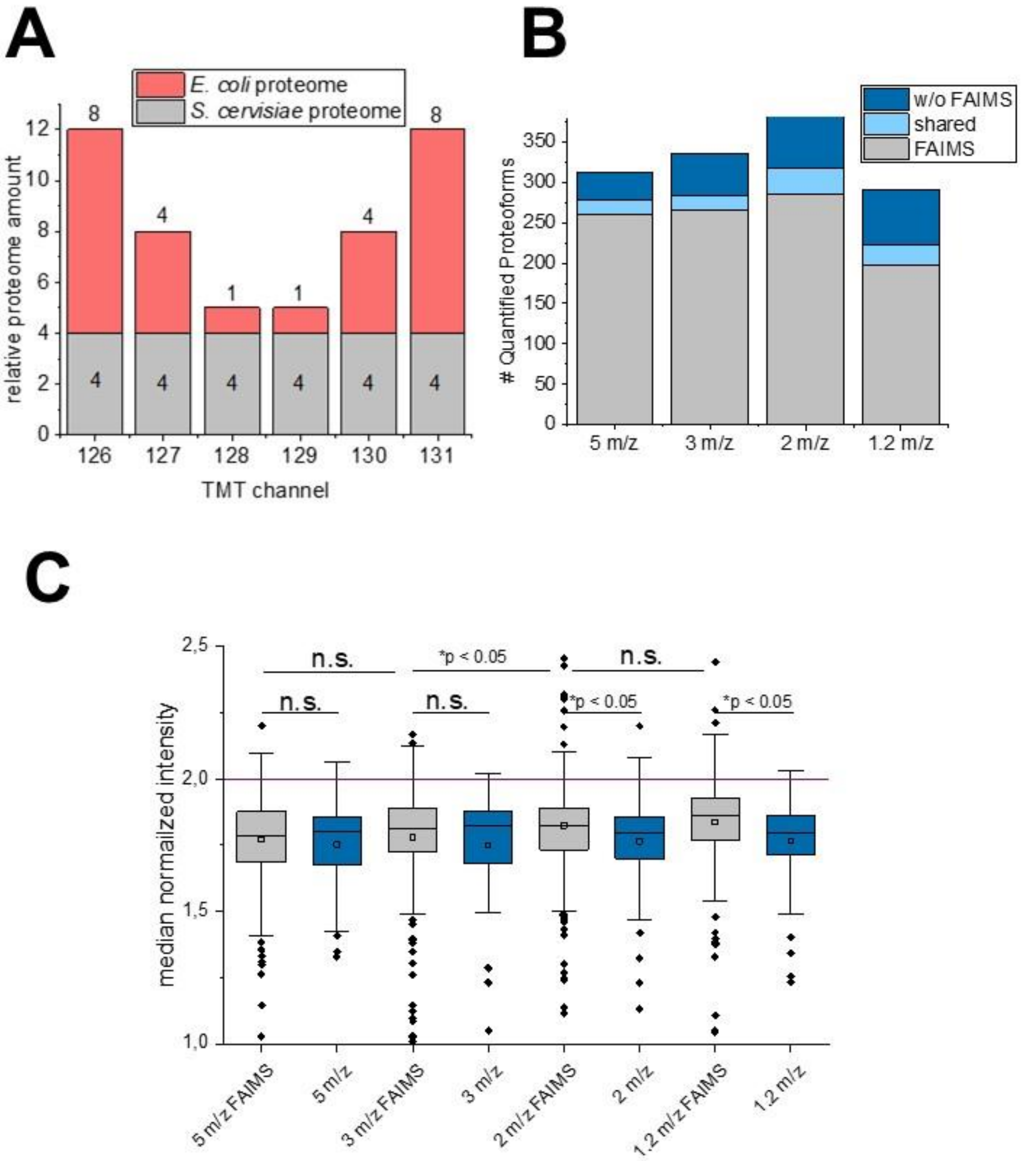

Suppl. Fig. 4: A TMT interference dataset was generated to assess ratio compression and coisolation effects. (A) A two-species mix consisting of *E. coli* proteome (8:4:1:1:4:8) and *S. cerevisiae* proteome (4:4:4:4:4:4:4) was labeled with iodoTMT sixplex. Evaluation of the influence of FAIMS and the isolation window on (B) the number of quantified proteoforms and (C) the quantitative accuracy. The purple line indicates the true value.

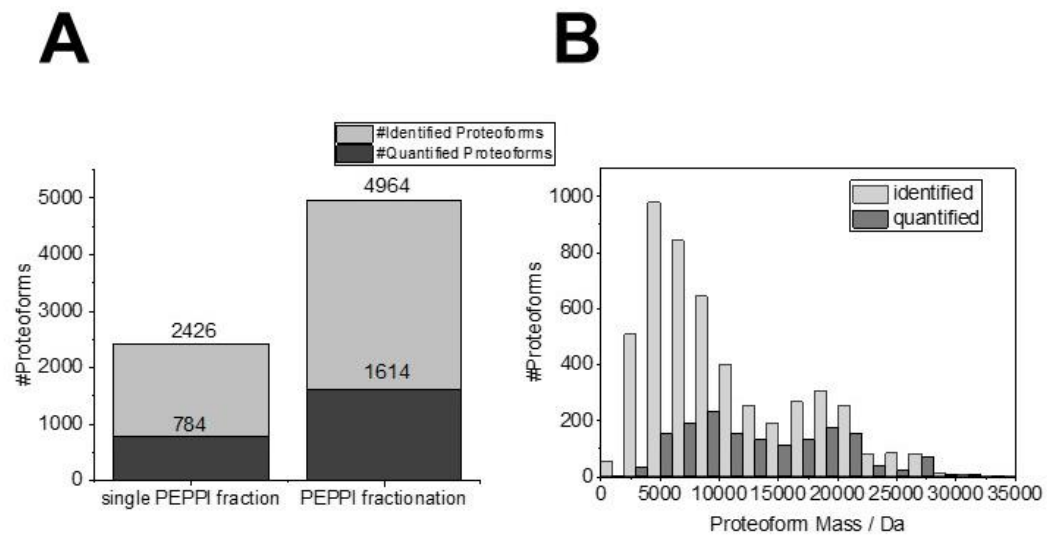

Suppl. Fig. 5: Using ten PEPPI fractions provided additional depth to the quantitative TDP analysis of *E. coli*. (A) Number and (B) mass distribution of identified and quantified proteoforms.

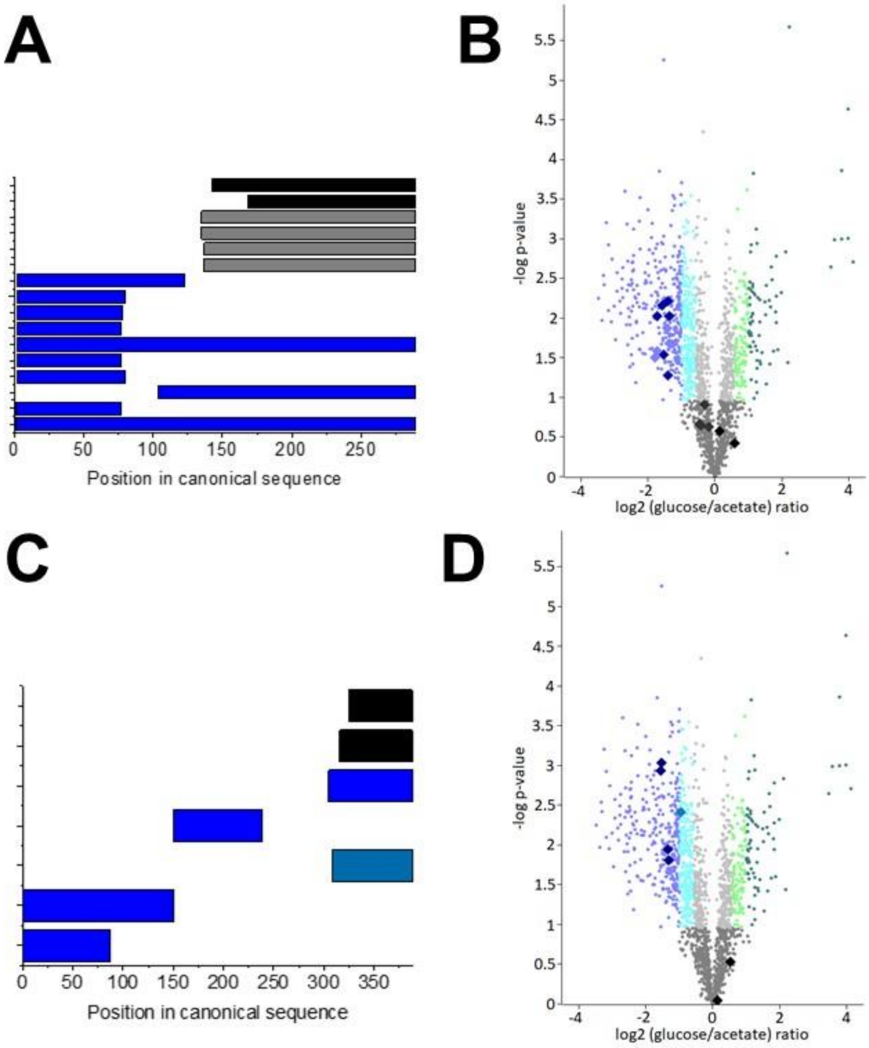

Suppl. Fig. 6: A total of 16 proteoforms were identified for the succinate-CoA ligase [ADP-forming] subunit alpha. Proteoforms are colored according to their abundance in the compared growth conditions: blue = proteoforms that were significantly higher abundant under acetate growth conditions (dark blue for log2-fold change >1, light blue for log2-fc 0.585-1); black = proteoforms below the 5% FDR cutoff of the t-test. All proteoforms with a t-test FDR between 1-5% with a log2 fc smaller than |0.585| are displayed in gray. All C-terminally truncated proteoforms (A) were significantly higher abundant under acetate growth conditions (B). All N-terminally truncated proteoforms, except one, were not differentially abundant. The proteoforms are highlighted in the same colors in (A) and (B). (C, D) Seven proteoforms of the succinate-CoA ligase [ADP-forming] subunit beta were quantified. Two C-terminally truncated proteoforms (C) were significantly higher abundant under acetate growth conditions (D). The coloring of individual proteoforms was kept the same in (C) and (D).

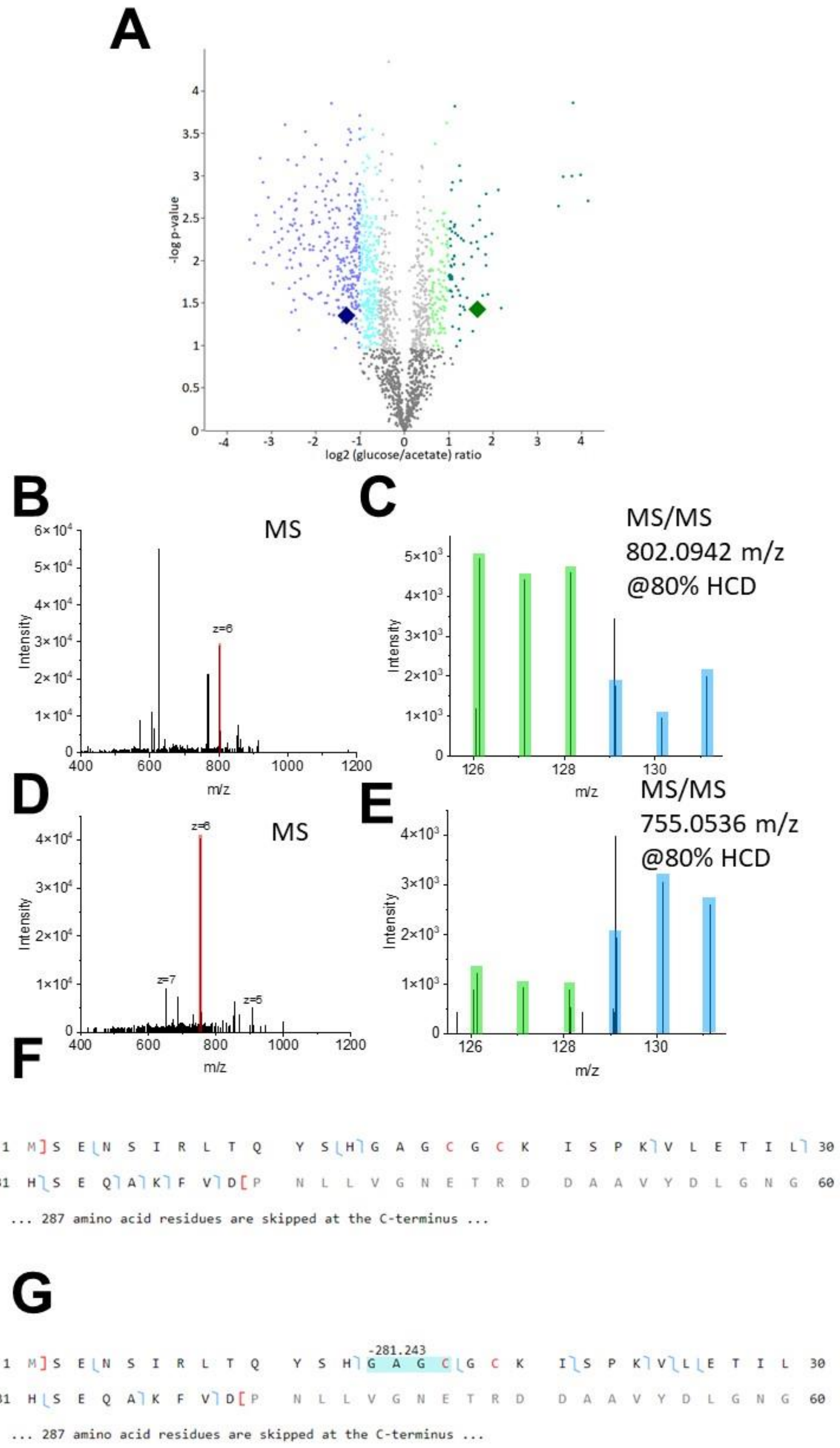

Suppl. Fig. 7: Two proteoforms with the same canonical sequence of the selenide, water dikinase were found to be significantly differentially abundant (A) both under different growth conditions. Under glucose growth conditions (highlighted in green), the unmodified proteoform (B,C,F) was higher abundant. The respective TMT channels are highlighted in the same colors as in (A). Under acetate growth conditions (highlighted in blue), a proteoform with a mass shift of -281.243 Da was significantly higher abundant (D,E,F); in this proteoform, labeling of Cys17 was potentially blocked by tri-oxidation of this residue.

References

(1) Scheer, J. M.; Ryan, C. A. A method for the quantitative recovery of proteins from polyacrylamide gels. *Anal. Biochem.* **2001**, *298*, 130–132.

(2) Nishida, H.; Kanao, E.; Ishihama, Y. Centrifugal Gel Crushing Tips for Gel-Based Proteome Analysis. *Anal. Chem.* **2023**, *95*, 18311–18315.

(3) Lazarev, A. V.; Rejtar, T.; Dai, S.; Karger, B. L. Centrifugal methods and devices for rapid in-gel digestion of proteins. *Electrophoresis* **2009**, *30*, 966–973.

(4) Jeong, K.; Kim, J.; Gaikwad, M.; Hidayah, S. N.; Heikaus, L.; Schlüter, H.; Kohlbacher, O. FLASHDeconv: Ultrafast, High-Quality Feature Deconvolution for Top-Down Proteomics. *Cell Systems* **2020**, *10*, 213-218.e6.

(5) Kaulich, P. T.; Winkels, K.; Kaulich, T. B.; Treitz, C.; Cassidy, L.; Tholey, A. MSTopDiff: A Tool for the Visualization of Mass Shifts in Deconvoluted Top-Down Proteomics Data for the Database-Independent Detection of Protein Modifications. *J Proteome Res.* **2022**, *21*, 20–29.

Supplementary Tables

Suppl. Table 1: MS and FAIMS settings used in this study. The one-scan methods used a single scan for simultaneous proteoform identification and quantification, while the two-scan methods used two independent scans for proteoform identification and quantification. Since two independent MS2 fragmentation scans are used for the two-scan methods, two different MS2 settings are listed in the table.

MS setting	two scan high/high	two scan medium/high	one scan high/high	one scan medium/high
Application mode	peptide mode	peptide mode	peptide mode	peptide mode
Compensation voltages	-60 ,-50 ,-40 ,-20 V	-20, 0, 15, 30 V	-60 ,-50 ,-40 ,-20 V	-20, 0, 15, 30 V
MS1 resolution	-60, -50 V: 60k	7.5k	-60, -50 V: 60k	7.5k
	-40, -20 V: 120k		-40, -20 V: 120k	
MS1 normalized AGC target	200%	200%	200%	200%
MS1 max. injection time	246 ms	50 ms	246 ms	50 ms
MS1 number of microscans	-60, -50 V: 2	10	-60, -50 V: 2	10
	-40, -20 V: 4		-40, -20 V: 4	
MS2 resolution	60k	60k	60k	60k
MS2 normalized AGC target	400%	400%	400%	400%
MS max. injection time	250 ms	250 ms	250 ms	250 ms
MS2 number of microscans	-60, -50 V: 2	-20, 0 V: 2	-60, -50 V: 2	-20, 0 V: 2
	-40, -20 V: 4	15, 30 V: 4	-40, -20 V: 4	15, 30 V: 4
Activation type	CID, 25%	CID, 25%	HCD, stepped (30, 40, 50%)	HCD, stepped (30, 40, 50%)
Isolation window	2 m/z	2 m/z	2 m/z	2 m/z
Scan range	auto	Auto	120-1800 m/z	120-1800 m/z
MS2 resolution	60k	60k		
MS2 normalized AGC target	400%	400%		
MS max. injection time	118 ms	118 ms		
MS2 number of microscans	2	2		
Activation type	HCD, 80%	HCD, 80%		
